# 1-(4-Bromo­phen­yl)-3-(4-ethoxy­phen­yl)­prop-2-en-1-one

**DOI:** 10.1107/S1600536808021776

**Published:** 2008-07-19

**Authors:** Hoong-Kun Fun, P. S. Patil, S. M. Dharmaprakash, Suchada Chantrapromma

**Affiliations:** a X-ray Crystallography Unit, School of Physics, Universiti Sains Malaysia, 11800 USM, Penang, Malaysia; b Department of Studies in Physics, Mangalore University, Mangalagangotri, Mangalore 574 199, India; c Crystal Materials Research Unit, Department of Chemistry, Faculty of Science, Prince of Songkla University, Hat-Yai, Songkhla 90112, Thailand

## Abstract

The title compound, C_17_H_15_BrO_2_, consists of two substituted benzene rings connected by a prop-2-en-1-one group. The mol­ecule is nearly planar and adopts an *E* configuration. The dihedral angle between the two benzene rings is 8.51 (19)°. The enone plane makes dihedral angles of 11.06 (19) and 7.69 (19)°, respectively, with the bromo­phenyl and ethoxy­phenyl rings. The mol­ecules are linked by C—H⋯O hydrogen bonds to form a zigzag ribbon-like structure along the *b* direction. The crystal structure is stabilized by weak intra- and inter­molecular C—H⋯O inter­actions.

## Related literature

For hydrogen-bond motifs, see: Bernstein *et al.* (1995 ▶). For similar structures, see: Fun *et al.* (2008 ▶); Patil, Fun *et al.* (2007 ▶); Patil, Ng *et al.* (2007 ▶). For background on chalcones, see: Chopra *et al.* (2007 ▶); Fichou *et al.* (1988 ▶); Goto *et al.* (1991 ▶); Gu, Ji, Patil & Dharmaprakash (2008 ▶); Gu, Ji, Patil, Dharmaprakash & Wang (2008 ▶); Sathiya Moorthi, Chinnakali, Nanjundan, Radhika *et al.* (2005 ▶); Sathiya Moorthi, Chinnakali, Nanjundan, Selvam *et al.* (2005 ▶); Schmalle *et al.* (1990 ▶); Uchida *et al.* (1998 ▶); Wang *et al.* (2004 ▶); Zhao *et al.* (2000 ▶).
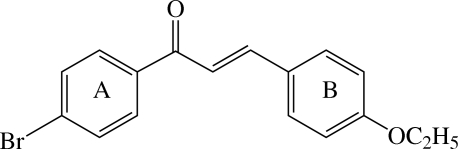


## Experimental

### 

#### Crystal data

C_17_H_15_BrO_2_
*M* *_r_* = 331.19
Monoclinic, 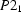

*a* = 3.9855 (1) Å
*b* = 10.0681 (3) Å
*c* = 17.5270 (4) Å
β = 92.227 (2)°
*V* = 702.77 (3) Å^3^
*Z* = 2
Mo *K*α radiation
μ = 2.92 mm^−1^
*T* = 100.0 (1) K
0.47 × 0.17 × 0.09 mm

#### Data collection

Bruker SMART APEXII CCD area-detector diffractometer
Absorption correction: multi-scan (*SADABS*; Bruker, 2005 ▶) *T*_min_ = 0.340, *T*_max_ = 0.781
7696 measured reflections
2620 independent reflections
2339 reflections with *I* > 2σ(*I*)
*R*_int_ = 0.033

#### Refinement

*R*[*F* ^2^ > 2σ(*F* ^2^)] = 0.033
*wR*(*F* ^2^) = 0.095
*S* = 1.08
2620 reflections
182 parameters
1 restraint
H-atom parameters constrained
Δρ_max_ = 0.98 e Å^−3^
Δρ_min_ = −0.53 e Å^−3^
Absolute structure: Flack (1983 ▶), 656 Friedel pairs
Flack parameter: 0.013 (13)

### 

Data collection: *APEX2* (Bruker, 2005 ▶); cell refinement: *APEX2*; data reduction: *SAINT* (Bruker, 2005 ▶); program(s) used to solve structure: *SHELXTL* (Sheldrick, 2008 ▶); program(s) used to refine structure: *SHELXTL*; molecular graphics: *SHELXTL*; software used to prepare material for publication: *SHELXTL* and *PLATON* (Spek, 2003 ▶).

## Supplementary Material

Crystal structure: contains datablocks global, I. DOI: 10.1107/S1600536808021776/fl2206sup1.cif

Structure factors: contains datablocks I. DOI: 10.1107/S1600536808021776/fl2206Isup2.hkl

Additional supplementary materials:  crystallographic information; 3D view; checkCIF report

## Figures and Tables

**Table 1:** Hydrogen-bond geometry (Å, °)

*D*—H⋯*A*	*D*—H	H⋯*A*	*D*⋯*A*	*D*—H⋯*A*
C4—H4*A*⋯O2^i^	0.93	2.57	3.257 (5)	131
C9—H9*A*⋯O1	0.93	2.48	2.814 (5)	102
C16—H16*B*⋯O1^ii^	0.97	2.49	3.446 (5)	170

Symmetry codes: (i) 
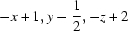
; (ii) 
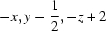
.

**Fig. 1.**
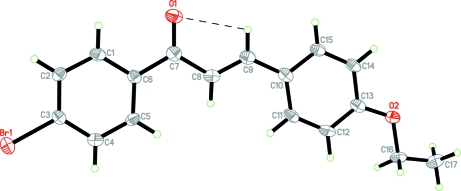
The molecular structure of (I) showing 50% probability displacement ellipsoids and the atom-numbering scheme. The dashed line represents the intra-molcular C—H···O interaction.

**Fig. 2.**
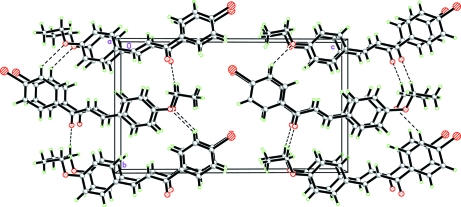
Crystal packing for (I) viewed along the a axis showing an antiparallel arrangement of the molecules. Hydrogen bonds are shown as dashed lines.

**Fig. 3.**
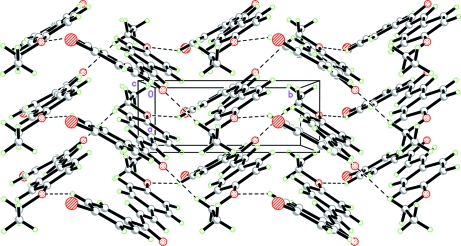
Crystal packing for (I) showing the zigzag ribbon-like structure running along the b axis. Hydrogen bonds are shown as dashed lines.

## supplementary crystallographic information

### Comment

Chalcone and its derivatives have received much attention due to their interesting biological (Nel *et al.*, 1998) and non-linear optical properties (Chopra *et al.*, 2007; Sathiya Moorthi, Chinnakali, Nanjundan, Radhika *et al.*, 2005; Sathiya Moorthi, Chinnakali, Nanjundan, Selvam *et al.*, 2005; Schmalle *et al.*, 1990; Wang *et al.*, 2004; Gu, Ji, Patil & Dharmaprakash, 2008; Gu, Ji, Patil, Dharmaprakash & Wang, 2008). Understanding the origin and magnitude of nonlinearity in such exotic molecules is very important from both a fundamental point of view and for its wide range of applications. Some chalcone derivatives exhibiting second harmonic generation (SHG) also possess other attributes such as transparency in the relevant wavelengths, ability to withstand laser irradiation, and chemical stability (Fichou *et al.*, 1988; Goto *et al.*, 1991; Uchida *et al.*, 1998; Zhao *et al.*, 2000). We previously reported the crystal structure of a related chalcone derivative, 1-(3-bromophenyl)-3-(4-ethoxyphenyl) prop-2-en-1-one, (II) (Fun *et al.*, 2008). In our continuing systematic study, we report here the structure of the title compound, (I) which also crystallized in a non-centrosymmetric space group and, as with (II), it should exhibit second-order nonlinear optical properties.

The molecular structure of (I) (Fig. 1) consists of two planar six-membered rings C1–C6 (ring *A*) and C10–C15 (ring *B*), with maximum deviations of 0.009 (4) and -0.010 (4) Å for atoms C6 (ring A) and C12(ring B), respectively. The molecule exists in an *E* configuration with respect to the C8=C9 double bond [1.336 (6) Å]: the torsion angle C7–C8–C9–C10 = -179.4 (4)°. The molecule is nearly planar with a dihedral angle between rings *A* and *B* of 8.51 (19)° [10.09 (11)° in (II) by Fun *et al.*, 2008]. The mean plane *C* through enone unit (C7–C9/O1) makes dihedral angles of 11.06 (19)° and 7.69 (19)° with the planes of rings *A* and *B*, respectively [the corresponding values are 12.05 (11)° and 9.87 (11)° in (II)]. The ethoxy group itself is slightly twisted as indicated by the torsion angle C13—O2—C16—C17 = 173.4 (4)° but co-planar with the attached benzene ring *B* with the torsion angle C16/O2/C13/C12 = -1.2 (6)°. A weak C9–H9A···O1 intramolecular interaction (Fig. 1) generates an S(5) ring motif (Bernstein *et al.*, 1995). The overall conformation of (I) is flatter than that observed for (II) which can be attributed to the different positions of the Br substituent on ring *A* (*para* in (I) and *meta* in (II). The bond distances and angles in (I) have normal values and are comparable with a number of closely related structures (Fun *et al.*, 2008; Patil, Fun *et al.*, 2007; Patil, Ng *et al.*, 2007).

In the crystal packing, the molecules are arranged in an anti-parallel manner and linked by weak C—H···O interactions (Table 1) into a zigzag ribbon-like structure along the *b* direction (Fig. 2 and Fig. 3). Similar packing characteristics were observed in (II) (Fun *et al.*, 2008). In (I) the same weak C—H···O (C16—H16B···O1) interaction is involved in the ribbon-linkage but there is also an additional weak C—H···O interaction which links the molecules (Table 1). This is also due to the different positions of the *meta* and *para* Br substitutions in (I) and (II) which made (I) more favourable for the C—H···O contacts.

### Experimental

The title compound was synthesized by the condensation of 4-ethoxybenzaldehyde (0.01 mol, 1.39 ml) with 4-bromoacetophenone (0.01 mol, 1.99 g) in methanol (60 ml) in the presence of a catalytic amount of sodium hydroxide solution (5 ml, 20%). After stirring for 3 h, the contents of the flask were poured into ice-cold water (500 ml) and left to stand for 4 h. The resulting crude solid was filtered and dried. Single crystals were obtained by recrystallization from acetone.

### Refinement

All H atoms were placed in calculated positions, with C—H = 0.93 Å, *U*_iso_ = 1.2*U*_eq_(C) for aromatic and CH, C—H = 0.97 Å, *U*_iso_ = 1.2*U*_eq_(C) for CH_2_ and C—H = 0.96 Å, *U*_iso_ = 1.5*U*_eq_(C) for CH_3_ atoms. A rotating group model was used for the methyl groups. The highest residual electron density peak is located at 0.78 Å from Br1 and the deepest hole is located at 0.84 Å from Br1.

### Crystal data

**Table:**

C_17_H_15_BrO_2_	*F*_000_ = 336
*M**_r_* = 331.19	*D*_x_ = 1.565 Mg m^−^^3^
Monoclinic, *P*2_1_	Mo *K*α radiation λ = 0.71073 Å
Hall symbol: P 2yb	Cell parameters from 2620 reflections
*a* = 3.9855 (1) Å	θ = 1.2–29.0º
*b* = 10.0681 (3) Å	µ = 2.92 mm^−^^1^
*c* = 17.5270 (4) Å	*T* = 100.0 (1) K
β = 92.227 (2)º	Block, colorless
*V* = 702.77 (3) Å^3^	0.47 × 0.17 × 0.09 mm
*Z* = 2	

### Data collection

**Table:**

Bruker SMART APEXII CCD area-detector diffractometer	2620 independent reflections
Radiation source: fine-focus sealed tube	2339 reflections with *I* > 2σ(*I*)
Monochromator: graphite	*R*_int_ = 0.033
Detector resolution: 8.33 pixels mm^-1^	θ_max_ = 29.0º
*T* = 100.0(1) K	θ_min_ = 1.2º
ω scans	*h* = −5→5
Absorption correction: multi-scan(SADABS; Bruker, 2005)	*k* = −13→8
*T*_min_ = 0.340, *T*_max_ = 0.781	*l* = −23→23
7696 measured reflections	

### Refinement

**Table:**

Refinement on *F*^2^	Hydrogen site location: inferred from neighbouring sites
Least-squares matrix: full	H-atom parameters constrained
*R*[*F*^2^ > 2σ(*F*^2^)] = 0.033	*w* = 1/[σ^2^(*F*_o_^2^) + (0.0562*P*)^2^] where *P* = (*F*_o_^2^ + 2*F*_c_^2^)/3
*wR*(*F*^2^) = 0.095	(Δ/σ)_max_ < 0.001
*S* = 1.08	Δρ_max_ = 0.98 e Å^−^^3^
2620 reflections	Δρ_min_ = −0.53 e Å^−^^3^
182 parameters	Extinction correction: none
1 restraint	Absolute structure: Flack (1983), 640 Friedel pairs
Primary atom site location: structure-invariant direct methods	Flack parameter: 0.013 (13)
Secondary atom site location: difference Fourier map	

### Special details

**Table:**

Experimental. The low-temperature data was collected with the Oxford Cyrosystem Cobra low-temperature attachment.
Geometry. All e.s.d.'s (except the e.s.d. in the dihedral angle between two l.s. planes) are estimated using the full covariance matrix. The cell e.s.d.'s are taken into account individually in the estimation of e.s.d.'s in distances, angles and torsion angles; correlations between e.s.d.'s in cell parameters are only used when they are defined by crystal symmetry. An approximate (isotropic) treatment of cell e.s.d.'s is used for estimating e.s.d.'s involving l.s. planes.
Refinement. Refinement of *F*^2^ against ALL reflections. The weighted *R*-factor *wR* and goodness of fit *S* are based on *F*^2^, conventional *R*-factors *R* are based on *F*, with *F* set to zero for negative *F*^2^. The threshold expression of *F*^2^ > σ(*F*^2^) is used only for calculating *R*-factors(gt) *etc*. and is not relevant to the choice of reflections for refinement. *R*-factors based on *F*^2^ are statistically about twice as large as those based on *F*, and *R*- factors based on ALL data will be even larger.

### Fractional atomic coordinates and isotropic or equivalent isotropic displacement parameters (Å^2^)

**Table:**

	*x*	*y*	*z*	*U*_iso_*/*U*_eq_	
Br1	0.43528 (8)	0.23538 (6)	0.502628 (18)	0.02637 (12)	
O1	−0.1514 (7)	0.6546 (3)	0.77360 (17)	0.0282 (7)	
O2	0.5201 (7)	0.5258 (3)	1.22900 (16)	0.0234 (6)	
C1	0.0563 (11)	0.5256 (4)	0.6436 (3)	0.0234 (9)	
H1A	−0.0604	0.6052	0.6379	0.028*	
C2	0.1400 (10)	0.4543 (4)	0.5789 (2)	0.0210 (8)	
H2A	0.0835	0.4859	0.5302	0.025*	
C3	0.3114 (9)	0.3340 (4)	0.5890 (2)	0.0195 (8)	
C4	0.3966 (9)	0.2855 (4)	0.6611 (2)	0.0229 (8)	
H4A	0.5097	0.2051	0.6669	0.027*	
C5	0.3112 (10)	0.3583 (4)	0.7242 (2)	0.0216 (8)	
H5A	0.3659	0.3258	0.7728	0.026*	
C6	0.1443 (10)	0.4796 (4)	0.7165 (2)	0.0197 (8)	
C7	0.0421 (11)	0.5603 (4)	0.7834 (2)	0.0241 (9)	
C8	0.1824 (10)	0.5255 (5)	0.8602 (2)	0.0249 (9)	
H8A	0.3356	0.4561	0.8655	0.030*	
C9	0.0927 (10)	0.5921 (4)	0.9221 (2)	0.0236 (9)	
H9A	−0.0626	0.6601	0.9142	0.028*	
C10	0.2115 (10)	0.5700 (4)	1.0008 (2)	0.0219 (8)	
C11	0.4006 (10)	0.4600 (4)	1.0240 (3)	0.0235 (9)	
H11A	0.4597	0.3979	0.9876	0.028*	
C12	0.5036 (10)	0.4399 (4)	1.0993 (2)	0.0241 (9)	
H12A	0.6252	0.3643	1.1133	0.029*	
C13	0.4243 (10)	0.5332 (4)	1.1541 (2)	0.0220 (8)	
C14	0.2317 (10)	0.6439 (4)	1.1322 (2)	0.0228 (9)	
H14A	0.1741	0.7059	1.1687	0.027*	
C15	0.1258 (10)	0.6625 (4)	1.0569 (2)	0.0223 (8)	
H15A	−0.0028	0.7365	1.0433	0.027*	
C16	0.7128 (11)	0.4120 (4)	1.2541 (3)	0.0247 (9)	
H16A	0.9075	0.4014	1.2231	0.030*	
H16B	0.5773	0.3322	1.2495	0.030*	
C17	0.8217 (13)	0.4350 (5)	1.3364 (3)	0.0328 (12)	
H17A	0.9605	0.3625	1.3541	0.049*	
H17B	0.6272	0.4407	1.3669	0.049*	
H17C	0.9465	0.5163	1.3406	0.049*	

### Atomic displacement parameters (Å^2^)

**Table:**

	*U*^11^	*U*^22^	*U*^33^	*U*^12^	*U*^13^	*U*^23^
Br1	0.02618 (18)	0.0284 (2)	0.02459 (17)	−0.0013 (3)	0.00238 (12)	−0.0061 (2)
O1	0.0305 (16)	0.0211 (17)	0.0332 (16)	0.0038 (13)	0.0033 (13)	−0.0008 (13)
O2	0.0222 (14)	0.0203 (15)	0.0275 (14)	0.0029 (12)	−0.0015 (12)	−0.0018 (12)
C1	0.026 (2)	0.014 (2)	0.030 (2)	−0.0016 (19)	0.0027 (18)	0.0004 (18)
C2	0.0227 (19)	0.019 (2)	0.0218 (18)	−0.0029 (17)	0.0000 (15)	0.0045 (15)
C3	0.0184 (17)	0.017 (2)	0.0229 (18)	−0.0049 (15)	0.0036 (14)	−0.0029 (15)
C4	0.0199 (17)	0.0192 (18)	0.029 (2)	0.0005 (17)	−0.0002 (15)	0.0007 (17)
C5	0.0247 (19)	0.017 (2)	0.0226 (19)	−0.0001 (16)	−0.0030 (16)	0.0040 (16)
C6	0.021 (2)	0.0155 (18)	0.0222 (18)	−0.0033 (15)	0.0012 (16)	−0.0020 (17)
C7	0.026 (2)	0.020 (2)	0.027 (2)	−0.0048 (17)	0.0057 (17)	0.0006 (17)
C8	0.0225 (19)	0.020 (2)	0.032 (2)	0.0018 (17)	0.0013 (17)	0.0015 (18)
C9	0.0233 (19)	0.018 (2)	0.029 (2)	−0.0030 (17)	0.0013 (16)	−0.0010 (17)
C10	0.0191 (18)	0.018 (2)	0.029 (2)	−0.0054 (16)	0.0046 (15)	−0.0038 (16)
C11	0.023 (2)	0.016 (2)	0.032 (2)	−0.0033 (16)	0.0084 (16)	−0.0057 (16)
C12	0.0209 (19)	0.0152 (19)	0.036 (2)	0.0016 (17)	0.0035 (17)	−0.0028 (18)
C13	0.0178 (18)	0.0172 (19)	0.031 (2)	−0.0035 (16)	0.0026 (16)	−0.0046 (17)
C14	0.0220 (19)	0.0169 (19)	0.030 (2)	−0.0018 (16)	0.0029 (16)	−0.0068 (17)
C15	0.0201 (18)	0.017 (2)	0.030 (2)	−0.0004 (15)	0.0011 (16)	−0.0030 (16)
C16	0.024 (2)	0.013 (2)	0.037 (2)	−0.0003 (16)	−0.0013 (18)	0.0026 (18)
C17	0.032 (3)	0.025 (2)	0.042 (3)	−0.002 (2)	−0.003 (2)	0.008 (2)

### Geometric parameters (Å, °)

**Table:**

Br1—C3	1.892 (4)	C9—C10	1.456 (6)
O1—C7	1.231 (5)	C9—H9A	0.9300
O2—C13	1.354 (5)	C10—C11	1.392 (6)
O2—C16	1.438 (5)	C10—C15	1.406 (6)
C1—C6	1.391 (6)	C11—C12	1.383 (6)
C1—C2	1.393 (6)	C11—H11A	0.9300
C1—H1A	0.9300	C12—C13	1.389 (6)
C2—C3	1.399 (6)	C12—H12A	0.9300
C2—H2A	0.9300	C13—C14	1.399 (6)
C3—C4	1.385 (6)	C14—C15	1.383 (6)
C4—C5	1.381 (6)	C14—H14A	0.9300
C4—H4A	0.9300	C15—H15A	0.9300
C5—C6	1.395 (6)	C16—C17	1.508 (6)
C5—H5A	0.9300	C16—H16A	0.9700
C6—C7	1.496 (6)	C16—H16B	0.9700
C7—C8	1.480 (6)	C17—H17A	0.9600
C8—C9	1.336 (6)	C17—H17B	0.9600
C8—H8A	0.9300	C17—H17C	0.9600
			
C13—O2—C16	117.9 (3)	C11—C10—C9	123.4 (4)
C6—C1—C2	121.1 (4)	C15—C10—C9	118.8 (4)
C6—C1—H1A	119.5	C12—C11—C10	122.2 (4)
C2—C1—H1A	119.5	C12—C11—H11A	118.9
C1—C2—C3	118.3 (4)	C10—C11—H11A	118.9
C1—C2—H2A	120.8	C11—C12—C13	119.6 (4)
C3—C2—H2A	120.8	C11—C12—H12A	120.2
C4—C3—C2	121.5 (4)	C13—C12—H12A	120.2
C4—C3—Br1	118.9 (3)	O2—C13—C12	124.7 (4)
C2—C3—Br1	119.6 (3)	O2—C13—C14	116.2 (4)
C5—C4—C3	119.0 (4)	C12—C13—C14	119.1 (4)
C5—C4—H4A	120.5	C15—C14—C13	121.0 (4)
C3—C4—H4A	120.5	C15—C14—H14A	119.5
C4—C5—C6	121.2 (4)	C13—C14—H14A	119.5
C4—C5—H5A	119.4	C14—C15—C10	120.3 (4)
C6—C5—H5A	119.4	C14—C15—H15A	119.8
C1—C6—C5	118.9 (4)	C10—C15—H15A	119.8
C1—C6—C7	118.2 (4)	O2—C16—C17	107.5 (4)
C5—C6—C7	122.8 (4)	O2—C16—H16A	110.2
O1—C7—C8	121.5 (4)	C17—C16—H16A	110.2
O1—C7—C6	119.8 (4)	O2—C16—H16B	110.2
C8—C7—C6	118.7 (4)	C17—C16—H16B	110.2
C9—C8—C7	121.1 (4)	H16A—C16—H16B	108.5
C9—C8—H8A	119.4	C16—C17—H17A	109.5
C7—C8—H8A	119.4	C16—C17—H17B	109.5
C8—C9—C10	127.2 (4)	H17A—C17—H17B	109.5
C8—C9—H9A	116.4	C16—C17—H17C	109.5
C10—C9—H9A	116.4	H17A—C17—H17C	109.5
C11—C10—C15	117.8 (4)	H17B—C17—H17C	109.5
			
C6—C1—C2—C3	0.9 (6)	C7—C8—C9—C10	−179.4 (4)
C1—C2—C3—C4	0.2 (6)	C8—C9—C10—C11	−10.6 (7)
C1—C2—C3—Br1	−179.1 (3)	C8—C9—C10—C15	170.8 (4)
C2—C3—C4—C5	−0.3 (6)	C15—C10—C11—C12	0.1 (6)
Br1—C3—C4—C5	179.0 (3)	C9—C10—C11—C12	−178.5 (4)
C3—C4—C5—C6	−0.6 (6)	C10—C11—C12—C13	−1.5 (6)
C2—C1—C6—C5	−1.8 (6)	C16—O2—C13—C12	−1.2 (6)
C2—C1—C6—C7	−179.2 (4)	C16—O2—C13—C14	178.8 (3)
C4—C5—C6—C1	1.6 (6)	C11—C12—C13—O2	−178.0 (4)
C4—C5—C6—C7	178.9 (4)	C11—C12—C13—C14	2.1 (6)
C1—C6—C7—O1	9.3 (6)	O2—C13—C14—C15	178.8 (4)
C5—C6—C7—O1	−168.0 (4)	C12—C13—C14—C15	−1.2 (6)
C1—C6—C7—C8	−169.7 (4)	C13—C14—C15—C10	−0.2 (6)
C5—C6—C7—C8	13.0 (6)	C11—C10—C15—C14	0.8 (6)
O1—C7—C8—C9	2.8 (7)	C9—C10—C15—C14	179.4 (4)
C6—C7—C8—C9	−178.3 (4)	C13—O2—C16—C17	173.4 (4)

### Hydrogen-bond geometry (Å, °)

**Table:**

*D*—H···*A*	*D*—H	H···*A*	*D*···*A*	*D*—H···*A*
C4—H4A···O2^i^	0.93	2.57	3.257 (5)	131
C9—H9A···O1	0.93	2.48	2.814 (5)	102
C16—H16B···O1^ii^	0.97	2.49	3.446 (5)	170

Symmetry codes: (i) −*x*+1, *y*−1/2, −*z*+2;  (ii) −*x*, *y*−1/2, −*z*+2.
